# School-based mental health screenings with Ukrainian adolescent refugees in Germany: Results from a pilot study

**DOI:** 10.3389/fpsyg.2023.1146282

**Published:** 2023-04-18

**Authors:** Claudia Catani, Jasmin Wittmann, Telja Lucia Schmidt, Sarah Wilker, Sina Neldner, Frank Neuner

**Affiliations:** Department of Psychology, Bielefeld University, Bielefeld, Germany

**Keywords:** Ukrainian war, refugees, stress, mental health, school-based screenings

## Abstract

Since the Russian invasion of Ukraine in February 2022, high numbers of Ukrainians, mostly women and children, have left the country. As of today, Germany has accepted more than one million refugees fleeing from Ukraine including ~200,000 children and adolescents registered in German schools. Since refugee minors are typically affected by high rates of mental health issues, the identification of potential psychological problems at an early stage after arrival is essential in order to make timely referrals for vulnerable youth to diagnostic or treatment services possible. The aim of the present study was to test the feasibility of a classroom-based mental health screening procedure and to assess symptoms of PTSD, depression, and anxiety in a small sample of adolescents who had fled to Germany. Forty-two adolescents (*n* = 20 girls) took part in the study. Screening results showed that more than half of the sample had elevated ratings in the Refugee Health Screener (RHS) and about 45% reported clinically significant levels of PTSD. Overall, the amount of both mental health problems and current worries related to the war was significantly higher in girls compared to boys. In general, screenings were well received by the adolescents. The findings of this pilot study point to a considerable level of mental health problems and distress in adolescent refugees affected by the recent war in Ukraine. Brief psychological screenings within the school setting might represent a promising approach to identifying potential mental health disorders as early as possible in newly arriving refugee youth.

## 1. Introduction

By the end of 2021, nearly 37 million children worldwide were displaced due to war, conflict and violence, including 13.8 million children seeking refuge and asylum in foreign countries. Not since the Second World War has the number of displaced minors been so high (United Nations, 2022), although people who fled the war in Ukraine are not yet included in this count. By the end of 2022, Germany alone has accepted more than one million refugees from Ukraine including ~200,000 children and adolescents registered in German schools (Mediendienst Integration, 2022).

So far, there are no reliable data on the consequences of the current war in Ukraine on the well-being of the affected children and adolescents. However, there is ample evidence on refugee minors from other conflict regions pointing to the detrimental effects of war trauma on mental health (Kadir et al., 2018; Müller et al., 2019; Dangmann et al., 2022) and on broader developmental outcomes that compromise social relations, school performance, and general life satisfaction (Catani, 2018). Posttraumatic Stress Disorder (PTSD) and depression are among the most common psychological disorders in child and adolescent refugees and asylum seekers with an average prevalence rate around 22% for PTSD and 14% for depression according to a recent meta-analysis (Blackmore et al., 2020).

It is reasonable to assume that many of the children and adolescents who have fled from Ukraine might show similar psychological problems as a result of the war, especially since large parts of the country’s population have already been suffering from a violent conflict for several years (Gonçalves Júnior et al., 2022). Additionally, in today’s digital world, even children who were able to escape the attacks on Ukraine in the early phase of the war often continue to experience the conflict indirectly through extensive media coverage in the news, through social media and continuous reports of family members who stayed behind in Ukraine (Elvevåg and DeLisi, 2022).

To prevent the development or deterioration of mental disorders in these young refugees, early referral to targeted diagnostic or treatment services following their arrival in the host country is necessary. However, given the high number of refugees in need, providing mental health services to this group is a challenge even for a country like Germany with a well-functioning health care system (Bajbouj, 2016; Nowak et al., 2022). To address this problem, the so-called “screen and treat” approach (Brewin et al., 2008), originally developed for the aftermath of disasters with high numbers of traumatized survivors, has been adapted to care for refugees in overburdened health systems. The first step in such an approach is to systematically conduct pragmatic mental health screenings with newly arrived refugees to identify those who are at risk of developing psychological disorders (Hollifield et al., 2016). To date, there are promising findings from studies with refugees in German reception centers showing that brief mental health screenings can be employed as a cost-effective measure to detect psychological problems thereby enabling targeted referrals to appropriate, evidence-based treatment services (Kaltenbach et al., 2017; Stingl et al., 2019). While the study by Stingl and colleagues only included adult refugees, the sample by Kaltenbach and colleagues comprised refugees over the age of 12, although the average age of the entire sample was still 29 (Kaltenbach et al., 2017). Overall, however, there is a lack of research regarding systematic mental health screenings with refugee minors in Germany as well as in other high-income host countries. This is unfortunate given that an early identification of psychological risks especially in this vulnerable group should be regarded as critical in order to prevent negative long-term mental health consequences.

To implement systematic screenings with refugee children and adolescents in Germany, schools may be an optimal setting since young refugees are admitted to schools rather quickly after arriving in Germany. In fact, schools have often been featured as key sites for the identification and treatment of mental health problems in non-refugee populations and there is evidence pointing to the effectiveness of mental health services delivered directly within the school setting (Stephan et al., 2015; Hoover and Bostic, 2021). In particular for refugee children, schools are an ideal place that can provide readily accessible mental health care, help with linguistic barriers and offer motivational support to strengthen adherence and participation in diagnostic and treatment (Fazel and Betancourt, 2018). Unfortunately, to date, there is little knowledge about the experience of refugee adolescents in the German educational system (Podar et al., 2022). In their qualitative study, based on interviews with teachers and school psychologists, Podar et al. concluded that schools in Germany have the potential to serve as the primary source of mental health support for refugee youth, but lack the resources to truly fulfill this role (Podar et al., 2022). Discussions with school psychologists pointed to a number of barriers that prevent refugee minors and their families from seeking help for mental health problems, such as a lack of knowledge about mental health care services and providers as well as fear of stigmatization.

Systematically conducting brief mental health screenings on every refugee minor who is newly admitted to school could therefore be an important step in improving access to mental health services and avoiding stigma.

Based on these considerations, the aim of the present pilot study was to determine the feasibility of a classroom-based mental health screening procedure for Ukrainian adolescents who had recently fled to Germany. The classroom as a screening setting is particularly suitable for young refugees from Ukraine, as so-called welcome classes were set up in many parts of Germany, in which pupils from Ukraine are initially taught separately. In addition, schools provide easy access to most families who have fled Ukraine. In fact, in a representative survey of Ukrainian refugees, conducted between August and October 2022, 91% of families with school-age children reported that at least one child attends school (Brücker et al., 2022).

Without claiming to determine the prevalence rate of mental disorders in a representative sample, we further aimed to gain an initial estimation of the mental health needs of adolescents who had recently fled the war in their home country and sought refuge in Germany. Given the scarcity of data on the well-being of Ukrainian children early after the flight, the assessment of mental health needs in an unselected convenience sample may allow to formulate targeted research questions and to propose appropriate diagnostic services for young refugees seeking asylum in Germany.

We used brief standardized self-report questionnaires to assess symptoms of PTSD, depression, and anxiety as well as questions addressing the amount of distress related to specific worries about the current war in Ukraine. Finally, we examined whether specific sociodemographic factors, i.e., age, gender, time since arrival in Germany, and the experience of war attacks prior to the flight are associated with the overall mental health burden reported by the adolescents.

## 2. Materials and methods

### 2.1. Sample

The present study was designed as a pilot school-based mental health screening with a convenience sample of adolescent refugees from Ukraine. The study was conducted in June 2022, ~4 months after the Russian invasion of Ukraine. In order to be eligible for this study, participants had to be aged over 16 so that they could give their consent to participate.

A total of 42 adolescents (20 girls) who fled their home country during the early phase of the war took part in the study. On average, participants had arrived in Germany 13 weeks ago. Table 1 provides an overview of participants’ sociodemographic characteristics. Since there were no significant differences between boys and girls for any of the reported variables, we have only included means and frequencies for the entire group of participants.

**Table 1:** Characteristics of participants (*N* = 42).

Variable	
Age, *M* (SD)	16.4 (0.73)
Gender, % (*n*) female	47.6 (*n* = 20)
Size of home town/place, % (*n*)	
<10,000	4.8 (2)
10,000–100,000	14.3 (6)
100,000–500,000	33.3 (14)
>500,000	47.7 (20)
Weeks in Germany (since flight), *M* (SD)	13.6 (3.2)
Current living situation, % (*n*)	
With relatives	7.3 (3)
With friends	2.4 (1)
Private housing/rent	70.7 (29)
Refugee center/group setting	19.5 (8)
Currently separated from family, % (*n*)	34.1 (14)
Home town/place was attacked before flight, % (*n*)	69.0 (29)

### 2.2. Procedure

Screenings were conducted at three different vocational schools in three so-called “welcome classes” attended only by young refugees from Ukraine. At the beginning of school lessons, the respective class was informed in detail about the contents and procedure of the screening by the study team. All adolescents present in the classes gave written informed consent and filled in the questionnaire.

During the screening procedure, a team of two psychologists and two interpreters with training and experience in translating in a mental health context were present in the room to assist the adolescents in case of questions or difficulties in understanding. The questionnaire took about 20 min to complete. After the screenings, the participants could take a short break during which the study team evaluated the questionnaires. Subsequently, every adolescent was offered a one-on-one consultation in which brief, individual feedback was provided. All but two of the participants agreed to take part in the personal feedback meeting that was conducted by a psychologist together with an interpreter.

Screenings were conducted as part of the recruitment procedure for a larger clinical trial with traumatized young refugees in Germany (Wilker et al., 2020; Wittmann et al., 2022). Out of the present sample, 11 children were invited for further detailed diagnostic interviews with the possibility of being treated within the controlled therapy study. As part of the ethical review of this trial, the procedure and the informed consent form of this screening were approved by the Ethic Committee of the German Psychological Association (Deutsche Gesellschaft für Psychologie, DGPs).

### 2.3. Instruments

We chose all instruments based on their psychometric properties, transcultural applicability, brevity, and previous use in (adolescent) refugee populations. All questionnaires were available in Russian prior to the start of our survey. We also prepared Ukrainian versions of the entire set of instruments, in case participants would feel more at ease with Ukrainian questions. However, when given the choice at the beginning of the screening, all participants chose to fill in the Russian questionnaires.

#### 2.3.1. Sociodemographic information

The first part of the screening questionnaire comprised a series of questions about individual sociodemographic characteristics, such as age, gender, place of origin as well as some basic information about the time of the flight and whether the hometown had been attacked before the flight. Also, adolescents were asked how often they followed the news about the war and the political situation in Ukraine.

#### 2.3.2. Mental health screening questionnaires

We included the Russian version of the Refugee Health Screener –15 (RHS-15; Hollifield et al., 2013) as a general measure of emotional distress in refugees aged 14 or older. The first 13 items of the RHS-15 assess symptoms of anxiety, depression, and trauma-related problems on a five-point Likert scale. Two additional items address a person’s level of functionality as well as the current distress level indicated by a distress thermometer ranging from 0 to 10. Whereas the first 14 items of the RHS-15 refer to the last month, the distress thermometer only refers to the last week (Hollifield et al., 2016). We used the cut off-score suggested by Hollifield et al. (2013) whereby a positive screening result is defined by a score of ≥12 on the items 1–14 or ≥5 on the distress thermometer. In the present study, the RHS-15 showed excellent internal consistency (*α* = 0.91). Three adolescents each had one missing response on the RHS. The missing values were replaced by imputed values, i.e., by the average value across all RHS items for the specific participant.

To identify participants with probable PTSD, the Primary Care PTSD Screen for DSM-5 (PC-PTSD-5; Prins et al., 2003) was used. The PC-PTSD-5 is a five-item dichotomous (yes/no) screening questionnaire measuring symptoms of PTSD over the past months. The total score is a count of the “yes” responses to the five questions about trauma-specific symptoms. The PC-PTSD showed acceptable internal consistency (*α* = 0.72) in the present study.

For three participants who each had missed one item, the missing value in the PC-PTSD was replaced by a mean imputation.

Depressive symptoms were measured by means of the Patient Health Questionnaire-9 (PHQ-9) which was originally designed to detect depression in primary care services (Spitzer et al., 1999; Kroenke and Spitzer, 2002). It comprises nine items that can be scored from 0 to 3, resulting in a total score between 0 and 27. A score ≥ 10 has been set as a cut-off for a potential major depressive disorder. The internal consistency of the PHQ-9 was good (*α* = 0.87) in the present sample.

The General Anxiety Disorder 7 (GAD-7) was originally developed to screen for general anxiety disorder in primary care (Spitzer et al., 1999, 2006) but has more recently been used to assess the severity of more generalized anxiety symptoms in refugee populations (Mitschke et al., 2013; Leiler et al., 2019). The questionnaire includes seven items that are scored from 0 to 3, resulting in a maximum total score of 21. We used a cut-off score ≥ 8, which has been suggested for a more general screening of anxiety symptoms (Kroenke et al., 2007). The GAD-7 showed good internal consistency (*α* = 0.84) in the present study.

#### 2.3.3. Questions about current worries related to the Ukrainian war

The following four self-developed items were used to ask about current concerns:

- How much do you worry about relatives or friends who are (still) in Ukraine?
- How much do you worry about your own educational and professional future?
- How much do you worry about the future of Ukraine?
- How much do thoughts of a possible expansion of the war or a third world war bother you?

Participants indicated their level of distress for each worry on a distress thermometer ranging from 0 (“not at all”) to 10 (“extremely worried”). The sum of the four items indicated the overall burden of concerns related to the war in Ukraine.

### 2.4. Data analysis

All statistical analyses were carried out using SPSS, Version 28 (IBM Corp., 2021) and JMP 8 (SAS Institute Inc., 2009). Group differences between boys and girls were analyzed by means of Mann–Whitney-*U* tests for continuous variables and Fisher’s exact tests for categorical variables. Correlations between the mean screening scores for the whole sample were calculated by means of Spearman rank correlations.

In order to identify specific factors associated with mental health problems, we conducted a linear regression analysis on the global mental health score including age, gender, time since arrival in Germany, and whether the home town had been attacked prior to the flight as predictor variables.

## 3. Results

### 3.1. Mental health outcomes and war-related worries

The majority of participants were affected by significant mental health problems, in particular by symptoms of posttraumatic stress. Table 2 provides an overview of the mean severity scores for the different screening instruments and the number of participants who scored above the cut-off values indicating potential mental health disorders. Considering all four domains of mental health that were assessed (general emotional distress, posttraumatic symptoms, depressive and anxious symptoms), 35.7% of the sample did not exceed the critical cut-offs in any domain whereas 21.4% exceeded the cut-off in only one domain. In total, 5 adolescents (11.9%) had critical ratings across two domains, 4 (9.5%) in three and another 21.4% of the sample (*n* = 9, 8 of them girls) exceeded the cut-off scores in all four screening tools.

**Table 2:** Results of the mental health screenings and comparison between boys and girls.

Mental health outcomes	Entire sample (*n* = 42)	Girls (*n* = 20)	Boys (*n* = 22)
RHS sum score, *M* (SD)	14.26 (10.79)	20.30** (10.63)	8.75 (7.65)
RHS above critical cut-off, % (*n*)	57.1 (24)	75.0* (15)	40.9 (9)
RHS distress thermometer >4, % (*n*)	21.5 (9)	45.0** (9)	0 (0)
PC-PTSD sum score, *M* (SD)	2.23 (1.59)	3.0** (1.59)	1.52 (1.33)
PC-PTSD above critical cut-off, % (*n*)	45.2 (19)	65.0* (13)	27.3 (6)
GAD-7 sum score, *M* (SD)	5.62 (4.05)	7.65** (4.39)	3.77 (2.65)
GAD-7 above critical cut-off, % (*n*)	33.3 (14)	55.0** (11)	13.6 (3)
PHQ-9 sum score, *M* (SD)	6.6 (5.7)	9.50** (6.09)	4.00 (3.86)
PHQ-9 above critical cut-off, % (*n*)	23.8 (10)	45.0** (9)	4.5 (1)

RHS, Refugee Health Screener 15; PC-PTSD, Primary Care PTSD Screen for DSM-5; GAD-7, General Anxiety Disorder 7; PHQ-9, Patient Health Questionnaire-9.

*Indicates significantly different means (Mann–Whitney *U*-test) and frequency distribution (Fisher’s Exact Test) for girls compared to boys, *p < 0.05, **p < 0.01.

As can be seen from Table 2, girls showed significantly higher scores compared with male participants on all mental health outcomes.

We found strong correlations between the different mental health screening outcomes for the entire sample. The RHS-15 was positively correlated with the PC-PTSD (*r_s_* = 0.73), the GAD-7 (*r_s_* = 0.87), and the PHQ-9 (*r_s_* = 0.80). In addition, there were positive correlations between the average score on the PC-PTSD and the GAD-7 (*r_s_* = 0.62) as well as the PHQ-9 (*r_s_* = 0.62). Finally, the mean score of the GAD-7 was strongly correlated with the PHQ-9 (*r_s_* = 0.88). All correlations were significant (*p* < 0.01).

A global mental health score was calculated as the mean of *z* transformed values of all mental health outcome measures that had been included in the screening, i.e., RHS-15, PC-PTSD, PHQ-9, and GAD-7.

**Figure 1:**
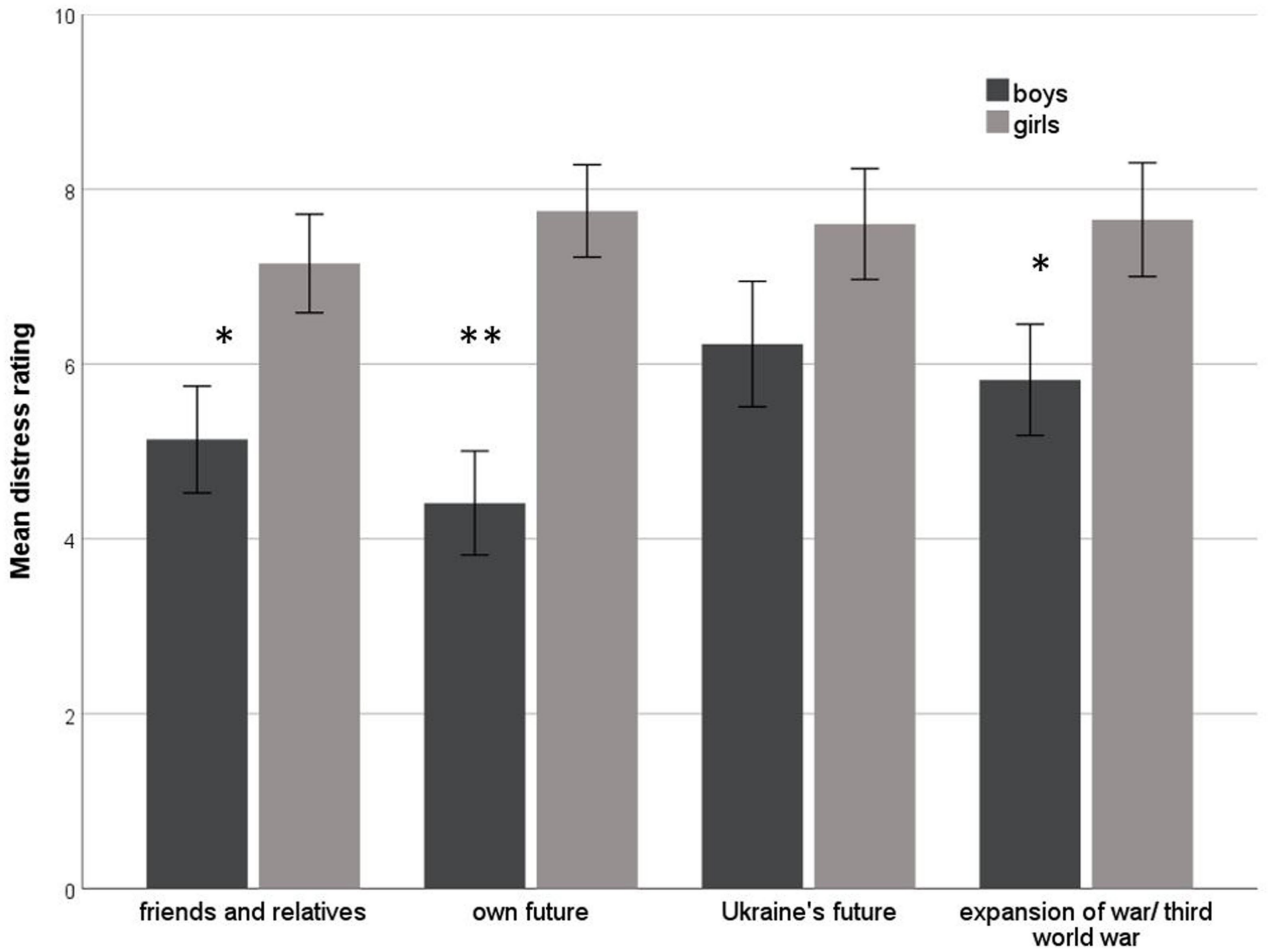
Distress ratings for worries related to the Ukrainian war. Means and standard errors are shown separately for girls and boys; * indicates significant group differences (Mann–Whitney *U*-test, *p < 0.05, **p < 0.01).

When asked about their current worries related to the war in Ukraine, the vast majority of the sample was found to be burdened by these worries. Again, girls had higher distress ratings in three out of four worry domains resulting in a significantly higher overall burden (*Mdn* = 25.4) compared to boys (*Mdn* = 17.9; *U* = 298.5, *p* < 0.05). Mean ratings and standard errors for current worries are shown in Figure 1.

Overall participants reported to follow the news about the situation in Ukraine on a very regular basis. 24.4% said they followed the news less than once per week, 24.4% stated they would search for updates on the war in their home country several times per week, 36.5% did so on a daily base, and 14.6% reported to have a look at the news almost every hour. There were no significant differences between boys and girls regarding their news seeking behavior.

### 3.2. Association between demographic factors and overall mental health problems

We calculated a linear regression model on the global mental health score (see Table 3). The model including age, gender, weeks since arrival in Germany, and whether the home town was attacked prior to the flight as predictors could explain almost 30% of the variance of the aggregated mental health score. Female gender was the only significant predictor for mental health problems in this model.

**Table 3:** Sociodemographic factors associated with mental health: standardized beta coefficients and zero-order correlation coefficients resulting from a linear regression model on global mental health score.

Predictor	*β*	Zero-order correlation
Age	0.22	−0.02
Gender (female)	0.59**	0.52**
Weeks since arrival in Germany	−0.18	−0.12
Home town attacked prior to flight (yes)	0.18	0.16

Full model’s adjusted *R*^2^ = 0.29; *F* (4.37) = 5.26, *p* < 0.01. Zero-order correlation is represented by Spearman’s rho for continuous predictor variables and point-biserial correlation for dichotomous predictor variables.

**Indicate associations significant at *p* < 0.01.

## 4. Discussion

By introducing classroom-based brief psychological screenings for Ukrainian refugee youth, the present pilot study demonstrated that this approach was feasible and well accepted by the participants. Overall, the screening procedure was easy to deliver, i.e., it took little time and required a manageable amount of human resources. For the most part, the students were able to complete the questionnaires on their own and only in rare cases did they need the help of the psychologists and translators present in the room. A remarkable result is that all but two of the adolescents took part in the brief individual consultation that was offered to every participant regardless of their mental health results. In fact, our impression was that the one-on-one interviews were indeed well accepted because they were presented as a regular part of the examination and did not only target those who showed signs of mental disorders. It can be assumed that such an approach is helpful to avoid possible fears of stigmatization among the young people.

Our finding regarding feasibility is in line with prior research on mental health screenings with non-refugee student populations (see review by Soneson et al., 2020). Regarding refugee children, research has focused primarily on school-based mental health interventions carried out in high-income countries without specifically examining the use of large-scale systematic psychological screenings in schools (see, for a review, Tyrer and Fazel, 2014; Sullivan and Simonson, 2016; Bennouna et al., 2019). As suggested by Podar et al. (2022), the possibility of low-threshold access to psychosocial care is rarely offered in German schools due to a lack of resources within the school system and various barriers making it difficult for refugee minors and their families to seek support (e.g., lack of knowledge about mental health care services, fear of stigmatization). Classroom-based mental health screenings ideally in combination with a brief psychoeducational session as proposed in the present pilot study, could therefore be an important step in overcoming these barriers and facilitating access to mental health services for adolescent refugees.

The Ukrainian adolescents examined here, too, seem to have an increased need for psychosocial support as more than 50% of the sample had elevated ratings in the RHS, 45% reported clinically significant levels of PTSD, and 33.3% and 23.8%, respectively, showed abnormal scores in anxiety and depression. Of course, since the present sample is small and far from being representative, these findings have to be treated with caution. However, they provide a first indication of increased vulnerability to mental illness among Ukrainian adolescents arriving in German schools. Overall, the elevated number of mental health problems found in this study is in line with a growing body of research on refugees from other war and crisis zones suggesting that refugee minors in Germany are at a high risk for developing mental health disorders, in particular PTSD and depression (Soykoek et al., 2017; Müller et al., 2019; Pfeiffer et al., 2019). It is important to note, though, that the assessment of psychological problems during such an early immigration phase as in the present study might lead to an overestimation of mental health disorders. Emotional distress in the first weeks after immigration may not necessarily be due to underlying mental illness, but may also be caused by current post-migration stressors (Li et al., 2016). In agreement with this, participants in the present study reported being very burdened by specific worries relating to the ongoing war in their home country and their personal future. In addition, more than one-third of the sample reported being currently separated from family, so anxiety about the well-being of family members who have stayed behind or who may be fighting in the war may serve to exacerbate mental health problems. A recent study with adult refugees found that standard cut-off values for screening tools such as the RHS lead to an over-estimation of the mental health need in the immediate aftermath of the flight and thus recommended that these cut-off values be increased in order to reach satisfying diagnostic specificity (Schmidt et al., 2022).

In the present study, the length of stay in Germany was not predictive of the global mental health score. This is in accordance with previous studies showing that mental health problems in refugee minors do not change considerably over time in the host country (Derluyn and Broekaert, 2007; Vervliet et al., 2014). However, the finding might also be attributed to the limited variance in this variable as all adolescents in the present study had fled Ukraine in the very early days or first weeks of the war. In the future, longitudinal studies are needed that follow young refugees from the first weeks after arrival in the host country over a longer period of time. Gender resulted as the only significant predictor in a regression model that could explain almost 30% of the variance. Strong gender effects were also noted with respect to all mental health outcomes with girls having significantly higher scores compared to boys on measures for PTSD, depression, anxiety, and emotional distress (RHS). Also, the amount of current worries about the war in Ukraine and one’s personal future was significantly higher in female participants. This gender effect is consistent with previous research pointing to a higher prevalence of depression (Piccinelli and Wilkinson, 2000) and PTSD (Hapke et al., 2006) in women compared to men, both for non-refugee populations as well as for asylum seekers and refugees (Tekin et al., 2016). A systematic review on gender differences in the mental health of unaccompanied refugee minors in Europe reported strong evidence for a higher burden of depression among girls and mixed evidence for an elevated PTSD symptom load in girls compared to boys (Mohwinkel et al., 2018). Since the brief screenings conducted in the present study were not designed to determine risk factors for mental health problems, it can only be speculated as to why the consistent gender effect was observed. It could simply reflect the typical pattern of PTSD and depression prevalences observed in the general population. In fact, previous research examining PTSD symptoms in internally displaced Ukrainians and refugees both before the recent Russian invasion (Shevlin et al., 2018) and after (Ben-Ezra et al., 2023) also point to dramatically higher PTSD rates in females. Differences in the amount and type (e.g., sexual violence, combat etc.) of experienced traumatic events might also account for the observed gender difference (Wilker et al., 2021); however, the present data cannot provide any insight into these mechanisms.

## 5. Conclusion

This pilot study aimed to evaluate the feasibility of brief classroom-based mental health screenings to assess symptoms of PTSD, depression, and anxiety in Ukrainian adolescents admitted to German schools. As already mentioned, a number of limitations have to be noted. The sample size is small and participants were recruited through selected schools and classes. Thus, findings cannot be generalized to the entire population of Ukrainian refugee minors in Germany. Moreover, the regression model analyzing potential predictors of mental health problems included only few variables and the assessment of war experiences in Ukraine was limited to one item, namely the question about an attack on the hometown. In order to actually identify risk factors associated with psychopathology, studies with much larger samples are needed, using instruments that allow quantification of different types of war events and other traumatic experiences. However, this aim goes beyond the scope of a brief mental health screening. Finally, as the present study did not include participants below the age of 16, conclusions about the psychological well-being of younger children who fled the Ukrainian war cannot be drawn. Since the requirement for parental consent has been shown to be a significant barrier to conducting school-based assessments of young children’s mental health (Soneson et al., 2020), future research should seek solutions to this problem.

Though the present results should be interpreted with cautions, they point to significant levels of mental health problems and distress due to worries among adolescent refugees affected by the recent war in Ukraine. Most importantly, our findings encourage the assumption that systematic mental health screenings of newly arriving refugees in schools may be a promising way to identify youth at risk of developing a mental disorder as early as possible. Such an approach should ideally be integrated with follow-up diagnostic and therapeutic services that are specifically tailored to the needs of these refugee minors in the early stages of their resettlement.

## Data availability statement

The raw data supporting the conclusions of this article will be made available by the authors upon request, without undue reservation.

## Ethics statement

The studies involving human participants were reviewed and approved by Ethic Committee of the German Psychological Association (Deutsche Gesellschaft für Psychologie, DGPs). The patients/participants provided their written informed consent to participate in this study.

## Author contributions

CC conceived and designed the study, participated in and supervised data acquisition, carried out statistical analyses, and drafted the manuscript. JW helped designing the study, trained and supervised interpreters, participated in data acquisition and critically revised the manuscript. TS and SW helped designing and coordinating the study, participated in data acquisition and critically revised the manuscript. SN helped designing the study, trained and coordinated interpreters and research assistants, supervised data entry and critically revised the manuscript. FN was the PI of the larger research project, conceived the study, and critically revised the manuscript. All authors contributed to the article and approved the submitted version.

## Funding

This study was part of the larger research project YOURTREAT funded by the German Federal Ministry of Education and Research (grant number 01GL1749C https://drks.de/search/de/trial/DRKS00017222). 

## Conflict of interest

The authors declare that the research was conducted in the absence of any commercial or financial relationships that could be construed as a potential conflict of interest.

## Publisher’s note

All claims expressed in this article are solely those of the authors and do not necessarily represent those of their affiliated organizations, or those of the publisher, the editors and the reviewers. Any product that may be evaluated in this article, or claim that may be made by its manufacturer, is not guaranteed or endorsed by the publisher.
